# Insights into the Mechanisms of Action of *Akkermansia muciniphila* in the Treatment of Non-Communicable Diseases

**DOI:** 10.3390/nu16111695

**Published:** 2024-05-29

**Authors:** Honorata Mruk-Mazurkiewicz, Monika Kulaszyńska, Wiktoria Czarnecka, Albert Podkówka, Natalia Ekstedt, Piotr Zawodny, Anna Wierzbicka-Woś, Wojciech Marlicz, Błażej Skupin, Ewa Stachowska, Igor Łoniewski, Karolina Skonieczna-Żydecka

**Affiliations:** 1 Department of Biochemical Science, Pomeranian Medical University in Szczecin, Broniewskiego 24, 71-460 Szczecin, Polandnatalia.ekstedt@gmail.com (N.E.); sanprobi@sanprobi.pl (I.Ł.); 2 Medical Center Zawodny Clinic, Ku Słońcu 58, 71-047 Szczecin, Poland; estetic@estetic.pl; 3 Sanprobi sp. z o.o. sp. k., ul. Kurza Stopka 5/c, 70-535 Szczecin, Poland; 4 Department of Gastroenterology, Pomeranian Medical University in Szczecin, Unii Lubelskiej, 71-252 Szczecin, Poland; 5 Department of Human Nutrition and Metabolomics, Pomeranian Medical University in Szczecin, Broniewskiego 24, 71-460 Szczecin, Poland

**Keywords:** postbiotic, novel food, microbiota

## Abstract

This comprehensive review delineates the extensive roles of *Akkermansia muciniphila* in various health domains, spanning from metabolic and inflammatory diseases to neurodegenerative disorders. *A. muciniphila*, known for its ability to reside in the mucous layer of the intestine, plays a pivotal role in maintaining gut integrity and interacting with host metabolic processes. Its influence extends to modulating immune responses and potentially easing symptoms across several non-communicable diseases, including obesity, diabetes, inflammatory bowel disease, and cancer. Recent studies highlight its capacity to interact with the gut–brain axis, suggesting a possible impact on neuropsychiatric conditions. Despite the promising therapeutic potential of *A. muciniphila* highlighted in animal and preliminary human studies, challenges remain in its practical application due to stability and cultivation issues. However, the development of pasteurized forms and synthetic mediums offers new avenues for its use in clinical settings, as recognized by regulatory bodies like the European Food Safety Authority. This narrative review serves as a crucial resource for understanding the broad implications of *A. muciniphila* across different health conditions and its potential integration into therapeutic strategies.

## 1. Introduction

*Akkermansia muciniphila* (*A. muciniphila*) constitutes about 1% to 3% of the entire gut microbiota, with a concentration of 10^9^ colony-forming units per gram. This bacterium is abundantly present within the mucus layer of the intestine, enabling it to more readily interact with epithelial cells located at the tips of intestinal villi compared to bacteria that do not degrade mucin [1,2]. The bacterium is a Gram-negative obligatory anaerobe. Not only does it improve intestinal barrier integrity, but it also causes mucus degradation, using mucin as the main energy source [3,4]. However, by doing so, it can exert an inhibitory effect on the growth of pathogenic bacteria using the same energy source [5]. *A. muciniphila* (viable) was found to occupy niches in the intestinal mucus layer, which typically limited the space available for other mucin-degrading bacteria, as evidenced in piglets and mice infected with enterotoxigenic *Escherichia coli* (ETEC), the so-called antibiotic-induced microbiome-disordered (AIMD) model. Supplementation with *A. muciniphila* not only enhanced gut health but also reduced the abundance of harmful bacteria, thereby promoting a healthier gut microbiome composition [6].

*A. muciniphila* was first isolated and described in 2004 by the team of scientists from Wageningen University in the Netherlands led by Prof. Willem de Vos [1]. It was recognized as the first representative of *Verrucomicrobiota* (former name *Verrucomicrobia*) in the human intestine [4,7,8]. Moreover, in the last few years, other species belonging to the genus *Akkermansia* were also described, based on human fecal metagenomes datasets and isolated strains. As it was noticed, in samples derived from one host in 99%, only one particular *Akkermansia* sp. was identified, showing a mutual exclusion pattern. However, only the species *Akkermansia municiphila* was significantly negatively correlated with BMI [4].

*A. muciniphila* of low abundance is present in the small bowel, whilst the highest concentration is observed in the colon, particularly in the cecum. *A. muciniphila* abundance varies with a number of factors including age, with a general decrease with age [9,10], although overrepresentation of this bacterium has been reported in centenarians [11]. This can be due to unique dietary habits and lifestyles of centenarians that promote gut health [12] or genetic predispositions affecting immune responses, gut environment, and metabolism that could favor the maintenance of beneficial gut bacteria [13]. On the other hand, *A. muciniphila*, represents a taxonomic group of bacteria abundantly associated with healthy aging and subjected to loss in individuals with comorbidities associated with unhealthy aging [14].

*A. muciniphila* is implicated in various health conditions including metabolic health, obesity, diabetes, and inflammatory bowel disease (IBD). Its role is multifaceted, showing both potential benefits and complex interactions with the host’s health. The bacterium’s abundance in the gut has been linked to the state of glucose tolerance, insulin resistance, and type 2 diabetes (T2D), albeit with inconsistent findings across different studies, which may be attributed to the diversity in study populations and methodologies [15,16]. The potential health benefits of *A. muciniphila* stem from its capacity to degrade mucin, producing short-chain fatty acids (SCFAs) like acetate and propionate. These metabolites are known for their anti-inflammatory and metabolic effects, suggesting *A. muciniphila*’s role in weight regulation and immune modulation [17].

Interventions that alter the gut microbiota, such as calorie restriction or bariatric surgery, have been shown to impact *A. muciniphila* levels, correlating with improved metabolic health and reduced obesity. This underscores the importance of understanding the bacterium’s specific role in metabolic regulation and its potential as a therapeutic target [15].

Moreover, *A. muciniphila*’s action affects the intestinal barrier’s integrity, crucial in preventing conditions like chronic inflammatory diseases (IBD). It has been evidenced that *A. muciniphila* or its components, like the outer membrane protein Amuc_1100, can reduce inflammation and enhance the integrity of the intestinal barrier, highlighting its potential therapeutic role in IBD [18].

Despite such interesting potential, viable *A. muciniphila* could not find therapeutic application due to the instability of this bacterium resulting from its anaerobic nature. Cultivating and maintaining this anaerobic bacteria live is technically challenging and requires specific conditions that are difficult to replicate outside the gut environment [19]. Also, maintaining the viability of live *A. muciniphila* through the processes of production, storage, and administration is challenging. The bacterium’s sensitivity to oxygen and temperature changes makes it difficult to develop stable formulations that retain efficacy until they reach the consumer. To add, the necessity of using animal-derived components in its cultivation, which poses ethical and logistical challenges for large-scale production, is of concern [20]. At last, the use of live microorganisms in therapeutics is subject to stringent regulatory requirements [21].

Therefore, the crucial steps were the application of a synthetic medium for cultivation, followed by pasteurization at 70 °C. It turned out that pasteurization of *A. muciniphila* MucT cultivated on a synthetic medium increased its capabilities to limit the development of fat mass, insulin resistance, and dyslipidemia in experimental models [20].

On 7 July 2021, the European Food Safety Authority (EFSA) adopted a scientific opinion on the safety of pasteurized *A. muciniphila* as a novel food in accordance with Regulation (EU) 2015/2283 [22]. This opinion opened up numerous possibilities for the use of this form of bacteria as a “novel food”, which refers to food that was not consumed to a significant degree in the EU before 15 May 1997. Novel food is recently discovered food sources or food that has been newly developed, is innovative, has been produced using new technologies and manufacturing processes, or is traditionally consumed outside the EU but not within it [23]. For this reason, we decided to present current information on the potential application of pasteurized/viable *A. muciniphila* in various diseases, focusing on the mechanism of action and the results of experimental and clinical studies, using the narrative review format.

### Search Strategy

The methodology for this study was as for scoping review initially formulated by Arksey and O’Malley [24] with updates [25]. The five main phases were as follows: (1) Formulating the research question: “What is known about the *Akkermansia muciniphila* regarding its impact on non-communicable diseases?” A broad question was chosen to encompass as many facets of the topic as possible. (2) Identifying relevant studies: We searched PubMed and Google Scholar databases using key terms such as Akkermansia muciniphila AND (gut diseases OR psychiatric disease OR cancer OR metabolic disease OR neurodegenerative disease). Additionally, we manually reviewed references from relevant reviews discussing the overall impact of Akkermansia muciniphila on health. Studies in English were included without publication date restrictions but available by 1 January 2024. (3) Selecting studies: We included studies involving both animals and humans that provided data on the abundance of *Akkermansia muciniphia* in abovementioned health conditions or described a supplementation with either live or pasteurized *A. muciniphila* (or its components) to confer health benefits. The first and senior authors carried out the selection process over two weeks. (4) Charting the data: We extracted data on study protocols (disease type, intervention—if applicable and key outcomes). (5) Collating, summarizing, and reporting the results: We thematically organized the data according to preformulated types of diseases. We decided to add a paragraph on how to increase the abundance of *A. muciniphila* as this might be easily introduced in practice.

## 2. Modes of Action

Several mechanisms of action of *A. muciniphila* are highlighted in the literature that contribute to host health. In the case of pasteurized *A. muciniphila*, the primary mechanism involves improving the intestinal barrier function. A key role of the intestinal barrier is to stabilize the space between intestinal epithelial cells and fenestrae of gut endothelium in order to prevent the transmission of, among other things, bacterial fragments and toxins into the circulatory system [26]. The intestinal barrier also ensures the maintenance of normal mucus levels, which translates into both structural and functional stabilization of the intestinal microbiota [27]. *A. muciniphila* not only has the ability to degrade mucins, but also to stimulate their synthesis. Importantly, this process depends on the initial mucin thickness and intestinal eubiose [28]. Genome analyses allow to identify gene cluster encoding secretion proteins that very likely constitute the pili-like structures present on the *A. muciniphila* outer membrane and might be involved in crosstalk with the host [20]. One of the proteins named Amuc_1100 has been shown to modulate the intestinal barrier and its permeability by increasing the expression of genes encoding proteins such as occludin (Ocln) and claudin-3 (Cldn3) and cannabinoid receptor 1 (Cnr1). In addition, the bacterium has been shown to reduce lipopolysaccharide (LPS) synthesis [29,30]. What is also interesting is that recombinant Amuc_1100 supplementation examined in HFD-fed mice showed reduced body weight gain and plasma high-density lipoproteins and improved glucose tolerance. Gut barrier protein expression was greater than in rodents treated with A. *muciniphila* pasteurized cells [20]. Additionally, extracellular vesicles derived from *A. muciniphila* (AmEV) have been shown to reduce intestinal permeability by inducing AMPK activation [31,32]. To add, short chain fatty acids (SCFA), stimulated to be produced with the presence of *A. muciniphila*, as well as the Amuc_1100 protein, interact with FFAR2 and -3 and also the TLR2 receptors of goblet cells, thereby increasing their number and differentiation, which also translates into the amount of mucus produced. These phenomena improve the integrity of the intestinal barrier and also the renewal of the intestinal epithelium [33].

The evidence is mounting that *A. muciniphila* content is correlated with metabolic disease, but the molecular mechanisms of its exact effects on the host have not been fully defined. It was found that a mechanism standing behind the impact of *A. muciniphila* on metabolic health comes from a study in in C57BL/6J mice fed a high-fat diet (HFD) in which *A. muciniphila* was shown to increase thermogenesis and glucagon-like peptide-1 (GLP-1) secretion via inducing uncoupling protein in brown adipose tissue. Based on liquid chromatography and spectrophotometry, the researchers identified the protein that is secreted by *A. muciniphila* and causes these changes—the P9 protein. It interacts with the intercellular adhesion molecule 2 [5]. In addition, IL-6 deficiency abrogates the effect of P9 on glucose hemostasis and reduces the expression of ICAM-2 [34].

Another study found that one of the mechanisms of action of *A. muciniphila* in acute liver injury is the downregulation of pro-inflammatory cytokines (IL-2, INF-Y, IL12p40, and MCP-1). In addition, concomitant administration of live *A. muciniphila* (3 × 10^9^ CFU; 14 days) inhibited the overall infiltration of mononuclear leukocytes in the liver with immune-mediated damage and reduced TLR2 and TLR4 levels [35]. *A. muciniphila* was found to affect the low-density lipoproteins (LDL) receptor pathway, reducing apoB48 and apoB100 on the LDL lipoprotein [36]. By affecting the production of SCFA, *A. muciniphila* is involved in signal transduction through inhibition of histone deacetylase (HDAC) and activation of G protein-coupled receptors, which translates into stimulation of the immune system [29]. Furthermore, using lipidomic and metabolomic analysis in humans, Depommier et al. [37] found that administration of *A. muciniphila* MucT induces specific modulation of various bioactive lipids that have been identified as PPARα agonists (2-PG and 1-PG). Using untargeted metabolomic analysis, they were able to reconstruct a metabolic pathway indicating activation of fatty acid oxidation via β-oxidation, and all identified metabolites increased mitochondrial activity. It has been shown that pasteurized *Akkermansia* acts via the AMUC_1100 protein and the TLR2 receptor, making this action more controllable than that of a probiotic, which requires colonization and appropriate environmental conditions for its action. The AMUC_1000 protein improves intestinal barrier integrity and crosses the intestinal barrier in most likely microvesilces (MVs) and enters adipose tissue, where it induces lipolysis, inhibits lipogenesis, and promotes thermogenesis [38]. In addition, it increases mucus production, which is also an energy expenditure for the body. So, we have a triple mechanism of action that reduces inflammation, alters fat metabolism, and increases energy expenditure. Very importantly, pasteurization can possibly increase the bioavailability of AMUC_1100 by affecting the bacterial surface charge [39].

In addition, *A. muciniphila* has been shown to reverse the enzymatic expression of moonooxygenase 3 (FMO3) affecting the conversion of TMA to TMAO in the liver [20]. *A. muciniphila* administration (2 × 10^8^ CFU three times a week for 7 weeks) in diabetic mice (non-obese, diabetic mice; model for autoimmune type 1 diabetes) was also associated with increased expression of the antimicrobial peptide Reg3y associated with antimicrobial and anti-inflammatory activity. It increased anti-inflammatory macrophage levels and restored the number of Foxp3+ Treg regulatory cells in pancreatic islets as well [40]. It has also been shown that the Amuc_1100 protein can affect the expression of the enzyme Tph1, which regulates the rate of serotonin (5-HT) synthesis in RIN-14B cells, and it reduces the expression of the serotonin reuptake transporter (SERT) through interactions with TLR-2, resulting in improved 5-HT biosynthesis and its extracellular availability [18].

Overall, three major modes of action of *A. mucniphila* were identified: (i) improvement of gut barrier integrity, (ii) enhancement of metabolic health, and (iii) modulation of the immune system. Other mechanisms, including the role of *A. mucniphila*’s derived novel tripeptide RHK and its protective role in sepsis, have been described; however, they require more investigation for full elucidation [41].

**Figure 1:**
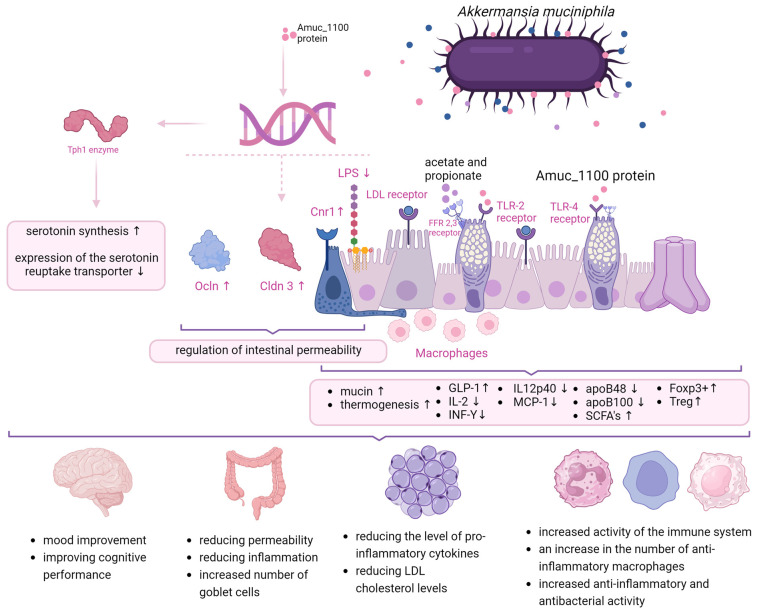
Modes of action of *A. muciniphila*. Tph1—tryptophan hydroxylase 1; Ocln—occludin, Cldn 3—claudin 3; Cnr1—cannabinoid receptor 1; LPS—lipopolysaccharide; TLR—Toll-like receptor; FFR—free fatty acid receptor; GLP—glucagon-like peptide; IL—interleukin; MCP—monocyte chemoattractant protein; Apo—apolipoprotein; Treg—regulatory T lymphocyte. Created with BioRender.

Figure 1 summarizes the major mechanisms of action described so far for *Akkermansia muciniphila*.

## 3. Gut Diseases

The impact of intestinal barrier permeability in IBD has been highlighted across many scientific studies [42]. Both animal and human studies have shown that these changes may translate into visceral sensitivity [43] with the heightened perception and responsiveness of the internal organs, particularly within the gastrointestinal (GI) tract to various stimuli [44].

### 3.1. Inflammatory Bowel Disease (IBD)

An association between increased intestinal barrier permeability resulting from tight junction alterations (e.g., an increase in the internalization of occludin and claudin-2; reduction of zonulin-1 and zonulin-2) and the development and progression of IBD has been documented. Also, the risk of developing IBD in healthy relatives suffering from Crohn’s disease has been associated with altered intestinal barrier permeability. *A. muciniphila* may have some potential in the treatment of IBD, though more research is necessary to fully understand its efficacy and mechanisms. In human studies, *A. muciniphila* abundance was shown to be significantly reduced in both ulcerative colitis (UC) and Crohn’s disease patients [33]. A study by Lo Sasso et al. compared changes in the gut microbiota in patients with IBD using 16S rRNA sequencing. A reduction in the abundance of *A. muciniphila* was observed in these patients [45]. Another study by Earley et al. compared changes in the composition of the gut microbiota in patients with active UC versus healthy patients or patients in remission of the disease. Patients with the active form of UC showed a significantly reduced abundance of *A. muciniphila* compared to other groups. The study confirmed an inverse relationship between *A. muciniphila* abundance and inflammation [46]. Lo Presti et al. compared the abundance of *A. muciniphila* in healthy individuals, patients with irritable bowel syndrome (IBS), and those with IBD. A significantly reduced abundance of *A. muciniphila* was observed in patients with IBD compared to the other groups [47]. Lopez-Siles et al. compared the content of *A. muciniphila* in healthy subjects versus patients with UC, Crohn’s disease, IBS, and colorectal cancer. Although no difference was observed in the study with regard to *A. muciniphila* abundance, its levels were reduced in subjects under 16 years of age with IBD compared to the control group [48]. The relationship between *A. muciniphila* abundance in pediatric patients struggling with Crohn’s disease was also investigated. In children and adolescents in remission, it was observed that the gut microbiota was abundant in *A. muciniphila* [49]. Therefore, the scientific data point towards a protective role for *A. muciniphila* in the course of IBD. The phenomenon behind this might be associated with a low amount of mucus in the gut of IBD patients—due to altered glycosylation or diminished sulphatation—limiting the bloom of *A. mucninphila* [46,50].

Also of note are studies in IBD patients, highlighting that fecal microbiota transplantation (FMT) led to increase in *A. muciniphila* in the gut of transplanted patients [51].

Studies using oral supplementation of *A. muciniphila* (1.5 × 10^8^ CFU) or the protein Amuc_1100 (3 µg) significantly reduced the infiltration of macrophages and CD8+ cytotoxic T lymphocytes in the colon of mice with colitis, resulting in a reduced inflammatory reaction [52]. Interestingly, administration of *A. muciniphila* (0.2 × 10^8^ CFU/mL; 4 weeks) attenuated neuroinflammation and enteric nervous system damage in Trinitro-benzene-sulfonic acid (TNBS)-induced chronic colitis [52,53]. Increased colonization of *Akkermansia muciniphila* (1 × 10^9^ CFU/mL live *A. muciniphila*) in the mouse intestine (CREBH knockout mice) elevated the expression of intestinal CREBH. This elevation was linked to the alleviation of endoplasmic reticulum stress, reduction of gut barrier permeability, and decrease in blood endotoxemia caused by dextran sulfate sodium (DSS) [54].

### 3.2. Irritable Bowel Syndrome (IBS)-like Symptoms in IBD Patients

Altered intestinal barrier permeability has also been observed in IBS patients with predominant diarrhea and in IBD patients in remission, manifesting IBS symptoms [55]. Of note, in the constipation type of IBS, the intestinal barrier is unaltered [44,56].

Irritable bowel syndrome (IBS) is a frequent disorder of gut–brain interaction and involves chronic symptoms without detectable inflammation or clearly visible damage in the digestive tract. However, IBD patients can frequently experience IBS-like symptoms, even when their colonic or intestinal inflammation is in remission. In patients with IBD who do not have active inflammation, the prevalence of IBS-like symptoms is ranging up to 41%, according to pooled data. This is higher than the prevalence of IBS in the general population, which ranges from 3% to 9% [57]. Whether the pathophysiology of IBS-like symptoms in IBD in remission is similar to that of IBS remains unknown. However, the persistence of symptoms has been associated with several features: (i) visceral hypersensitivity of enteric nervous system due to past-chronic inflammation; (ii) gut dysbiosis; (iii) post-inflammatory either anatomic or functional changes leading to altered bowel habits; and (iv) psychological factors, like stress and anxiety, which commonly exacerbate IBS-like symptoms.

Crohn’s disease patients are at a higher risk compared to those with ulcerative colitis, and females have a higher risk of developing IBS-like symptoms, reflecting broader trends seen in the general IBS population. Of interest, higher iron levels were associated with a decreased risk of IBS-like symptoms, though the mechanisms behind this are not fully understood [58].

Additionally, in the general population, the presence of an underlying medical condition such as IBD clinically precludes an IBS diagnosis. Therefore, the term “IBS-like symptoms in IBD in remission” currently best describes the symptom complexity in these patients.

IBS-like symptoms in IBD patients require a nuanced approach for diagnosis and management. IBS-like symptoms significantly impair the quality of life for IBD patients and can lead to increased healthcare utilization, higher rates of opioid use, and greater psychological distress. Therefore, tailoring treatments to the individual’s symptom profile and underlying pathophysiology is crucial for improving quality of life.

In addition to the use of *A. muciniphila* in the treatment of IBD, the use of postbiotic supplementation (pasteurized *A. muciniphila*) in irritable bowel syndrome (IBS)-like symptoms has recently received attention. Meynier et al. [59] aimed to investigate the effects of pasteurized *Akkermansia muciniphila* on alleviating IBS-like symptoms and related behavioral disorders in mice. The researchers used two mouse models to mimic IBS symptoms: (i) a non-inflammatory model to induce colonic hypersensitivity (NMS), and (ii) a *Citrobacter rodentium* post-infectious IBS (PI-IBS) model. Both models were treated with pasteurized *A. muciniphila*, and various parameters were evaluated, including gut permeability, colonic sensitivity, fecal microbiota composition, colonic IL-22 expression, anxiety-like behavior, and cognitive performance. The authors found that the presence of pasteurized *A. muciniphila* (6 × 10^8^ TFU of pasteurized *A. muciniphila* for 10 days) was associated with a significant (i) reduction in colonic hypersensitivity in both the NMS and PI-IBS models; (ii) improvement in the gut barrier function, as evidenced by lower FITC-dextran levels; and (iii) increase in expression of tight junction protein ZO-1 in the NMS and restored Claudin-2 levels in the PI-IBS models. Moreover, the study revealed improvement in anxiety-like behavior and memory defects and neuroinhibitory effects with reduced anxiety and enhanced cognitive function in the PI-IBS model treated with pasteurized *A. muciniphila.* Of interest, the treatment did not significantly alter the overall composition of the fecal microbiota, indicating that its beneficial effects might be due to direct interactions with the host rather than altering microbiota composition [59].

The summary of benefits and potential limitations of using *A. muciniphila* in gut diseases is presented in Table 1.

## 4. Metabolic Diseases

Recent studies have emphasized the link between damage to intestinal barriers and diseases, including diabetes, obesity, fatty liver, and cardiovascular disorders. It has been proposed that a weakened intestinal barrier may result in endotoxemia, which is connected to systemic inflammation, insulin resistance, diabetes, and lipid buildup, thereby hastening the progression of obesity and fatty liver diseases [60]. The pathologically increased permeability of the intestinal barrier is suspected to allow a significant influx of bacterial metabolites through the portal vein, causing the release of pro-inflammatory cytokines, chemokines, and eicosanoids. Both these metabolites and the substances released as a result of their presence induce a chronic inflammatory process and fibrosis of liver tissue, as well as having carcinogenic effects [61]. However, the exact processes by which intestinal barrier damage occurs and the methods to effectively enhance the intestinal barrier are still subjects for further investigation [60].

Numerous studies connecting the risk of type 1 diabetes with altered microbiota composition have pointed to higher abundance of *Bacteroides ovatus* and *Bacteroides uniformis*. In contrast *Bacteroides fragilis* abundance appears to play a protective role [62]. In healthy controls, butyrate-producing *Bifidobacteria* and mucin-degrading bacteria such as *A. muciniphila* make up a larger part of the microbiota composition, compared to patients with type 1 diabetes mellitus [63]. The protective potential of Bifidobacteria is not fully described, but de Goffrau’s study suggests that they limit the growth of *Bacteriodes* or restrict movement across the epithelium, thereby reducing inflammation [62]. However, Brown suggests that a microbiota rich in butyrate producers leads to increased amounts of mucin, tighter junctions, and increased intergranularity of the intestinal barrier, thereby creating an attractive environment for the proliferation of *A. muciniphila*, whose abundance is significantly higher in healthy individuals [63,64].

In type 2 diabetes, elevated levels of LPS are seen in patients with weight gain, insulin resistance, inflammation in the gut, and ultimately the progression of type 2 diabetes [65]. Elevated levels of LPS in some patients have also been associated with small intestinal bacterial overgrowth (SIBO) [66] and delayed gastrointestinal transit time [67]. A reduced abundance of *Faecalibacterium prausnitzii*, which are found in abundance in the microbiota of healthy individuals, was also noted in patients [68]. The intestines of patients with type 2 diabetes showed reduced numbers of Firmicutes and *Clostridia*. It has also been noted that the ratio of Bacteroidetes to Firmicutes is correlated with plasma glucose concentration [69].

It has been shown that disturbance of the intestinal microbiota can significantly affect liver health. Although the mechanism by which this would occur has not been sufficiently explored, a specific composition of the gut microbiota has been found in NAFLD patients [70].

Studies in mice have linked low intestinal abundance of *A. muciniphila* to pre-diabetes and diabetes [71]. Mice fed a high-fat diet (HFD) for 16 weeks showed increased expression of inflammatory markers, higher serum leptin levels, and development of hyperinsulinemia and hypoglycemia. After 3 weeks on the diet, the appearance of peripheral insulin resistance and a decrease in *A. muciniphila* counts were noted [72]. However, the reduced absolute bacterial count does not appear to affect only the development of pre-diabetic states in mice. In patients with developed type 2 refractory diabetes, manifested by a serum glycated hemoglobin level less than or equal to 8%, examination of fecal samples showed reduced numbers of *A. muciniphila* compared to patients who achieved optimal glycemic control with metformin or other hypoglycemic drugs [73].

In an experimental model of streptozotocin-induced type 2 diabetes in rats, live and pasteurized *A. muciniphila* significantly improved well-being, which was primarily associated with improved liver function, reduced plasma levels of pro-inflammatory factors, prevention of gluco/lipotoxicity, and reduction of oxidative stress. *A. muciniphila* at a concentration of 1 × 10^10^ CFU/mL was inactivated by pasteurization for 30 min at 70 °C for oral administration lasting 4 weeks [74].

An imbalance of the gut microbiota accompanies the development of both type II and type I diabetes, despite differences in the etiology of the two diseases [75]. In type I diabetes, it is IFN-γ that plays a key role in its pathogenesis. IFN-γ (−) mice show better glucose tolerance and increased numbers of *A. muciniphila* in the gut compared to wild-type mice. However, IFN-γ (−) mice without *A. muciniphila* did not show improved glucose tolerance This may indicate that the diabetogenic role of IFN-γ may be related to its ability to induce changes in the composition of the microbiota and, in particular, to reduce the abundance of *A. muciniphila* [76,77].

In addition, NOD mice (non-obese diabetic, mice with elevated fasting glucose levels) treated with oral vancomycin from 28 days of age showed a reduced incidence of type one diabetes, with *A. muciniphila* predominating in their microbiota. Furthermore, by observing two colonies of NOD mice, it was noted that a lower incidence of type one diabetes was always associated with an increased abundance of *A. muciniphila*. The above studies suggest a significant effect of *A. muciniphila* on the development of diabetes of both types, which may put forward the inclusion of a new therapy to combat the disease [40].

In the first of the human proof-of-concept clinical trials evaluating the validity of *A. muciniphila* supplementation, 40 overweight or obese subjects with insulin resistance were included. Patients received live or pasteurized *A. muciniphila* for 3 months. Primary endpoints included safety of the supplement and its tolerability (i.e., liver function, renal function, inflammation) and metabolic parameters (i.e., insulin resistance, circulating lipid concentrations, visceral fat, body mass index). Secondary endpoints included parameters of intestinal barrier function (i.e., plasma lipopolysaccharide (LPS)/metabolic endotoxemia), gut microbiota composition, and metabolites. After 2 weeks, the safety of the supplement was confirmed, as interpreted by the absence of changes in the measured biochemical parameters. Only a significant (still normal) reduction in GGT and CK activity in the group with pasteurized *A. muciniphila* was noted. There was also no significant change in the incidence of gastrointestinal disorders. After 3 months, a significant increase in fasting plasma insulin levels was observed in the placebo group (*p* < 0.05, T3 versus T0), in contrast to participants receiving both forms of *A. muciniphila*, who had reduced plasma insulin levels (by approximately 30%) compared with placebo. Insulin sensitivity was significantly reduced at T3 in the placebo group. Conversely, the supply of both forms of *A. muciniphila* improved this parameter. Pasteurized *A. muciniphila* clearly and significantly improved the insulin sensitivity index by approximately 30% compared to the placebo group, and live *A. muciniphila* significantly improved the insulin resistance index. Pasteurized *A. muciniphila* significantly reduced DPP-IV activity after 3 months of the study compared to baseline. WBC counts remained significantly increased compared to baseline and week 2 in the placebo group whereas supplementation with pasteurized *A. muciniphila* completely abolished this effect, resulting in a significant reduction in WBC counts compared to the placebo group. The magnitude of this difference between T0 and T3 or the placebo group (i.e., 866 cells/µL) is highly significant, as a difference of 300 to 1000 cells/µL is considered clinically significant. Supplementation with the pasteurized form of *A. muciniphila* significantly reduced both γGT and AST levels after 3 months compared to baseline. Specifically, γGT levels were markedly reduced by approximately 24% in the group supplemented with pasteurized *A. muciniphila* compared to T3 levels observed in placebo. Neither intervention induced a significant change in microbial community composition, although supplementation with live bacteria had a slightly greater effect (partial dbRDA, adjusted R^2^ = 0.03, *p* = 0.095) than pasteurized bacteria (partial dbRDA, adjusted R^2^ = 0.02, *p* = 0.14), while placebo had the least effect (partial dbRDA, adjusted R^2^ = 0.01, *p* = 0.66). Administration of pasteurized *A. muciniphila* significantly reduced total cholesterol by 8.68% compared with placebo, while LDL cholesterol was 7.53% lower and triglycerides 15.71% lower, although these differences did not reach statistical significance. The administration of pasteurized *A. muciniphila* slightly reduced body weight by about 2.27 kg, fat mass by about 1.37 kg, and hip circumference by 2.63 cm compared with the placebo group. Waist circumference decreased by approximately 1.56 cm. The study thus reports a broad-spectrum effect of *A. muciniphila* on changes associated with increased body weight [30]. Supplementation with *A. muciniphila* reverses metabolic disorders induced by a fatty diet, among others, metabolic endotoxemia, increased body fat, and insulin resistance. Administration of *A. muciniphila* decreased postprandial triglycerides and chylomicrons and increased expression of receptors for low-density lipoprotein (LDL), thereby regulating intermediate-density lipoprotein (IDL) through induction of apolipoprotein B 100 and apolipoprotein E [78,79,80].

*A. muciniphila* has the ability to activate immune signaling through receptors that recognize molecular patterns. There is increased secretion of the chemokine CCL20 binding to the CCR6 receptor on the surface of T and B lymphocytes. *A. muciniphila* has been shown to have a strong negative correlation with inflammatory markers, adipose tissue homeostasis, insulin levels, and glucose levels [5]. Supplementation-induced changes included stimulation of Treg cell proliferation and suppression of the hepatic stress marker ER-glucose-regulated protein [81]. In addition, *A. muciniphila* restored intestinal barrier function by reducing the high concentration of LPS induced by the Western diet. Consequently, this led to the alleviation of LPS-induced systemic inflammation and arthritis by reducing macrophage transmission to the inner membrane through reduced expression of TNF-α and IL-1β [82].

According to available data, *Akkermansia muciniphila* appears to have a positive effect on many metabolic disorders, but the exact mechanism of its action is not fully known. One of the causes of MAFLD (metabolic-dysfunction-associated fatty liver disease) is damage to the intestinal barrier with endotoxemia, which translates into the functioning of the microbiota–gut–hepatic axis. In the study by Wenrui Wu et al., the study mice were divided into three groups. Mice were fed a low-fat diet, a high-fat diet, or a high-fat diet with additional supplementation of approximately 1.5 × 10^9^ live *A. muciniphila* per day. It was shown that mice given *A. muciniphila* had less weight gain compared to the other groups. In addition, serum AST and ALT concentrations and NAS score (the NAS scale assesses the degree of liver fibrosis) were lower than in the group with the same diet without supplementation. Supplementation with *A. muciniphila* affects the gut microbiome in a way that alters the bile acid profile; improves glucose tolerance; reduces the development of white adipose tissue (WAT); and lowers insulin, resistin, and leptin levels [83]. In contrast, Sejeong et al. conducted a study in mice that were fed a diet consisting of 45% or 10% fat. Some of the individuals were also given *A. muciniphila* at a dose of 10^8^ to 10^9^ CFU/mL for 10 weeks. The researchers noted no differences in body weight gain between mice supplemented with *A. muciniphila* compared to mice without active supplementation. However, serum triglyceride and ALT levels were lower when *A. muciniphila* was introduced into the diet of intervention group, indicating it may reduce the liver damage caused by the disease. When the expression levels of chREBP and SREBP were determined in liver tissue, supplementation with *A. muciniphilia* decreased the expression levels of the genes tested, resulting in a reduction of triglyceride synthesis in the liver compared to mice that were not given the probiotic. The same relationship was found for IL-6. As mentioned earlier, the amount of *A. muciniphilia* decreases in individuals on a high-fat diet. The study showed that supplementation allows this effect to be reversed and the number of bacteria to increase. The researchers suspect that supplementation could improve the tightness of the intestinal barrier by maintaining homeostasis of the intestinal microbiome, thereby preventing the development of MAFLD [84]. Selected metabolic studies in which *A. muciniphila* supplementation was used are included in Table 2. The benefits of using *A. muciniphila* in metabolic diseases are found in Table 3.

## 5. Cancer

The state of the intestinal barrier is also important for the development of cancer in the body. It has been shown that excessive permeability within the intestinal barrier can lead to increased penetration of toxic and pro-inflammatory substances, which can then cause inflammation in the body and interfere with cell proliferation towards carcinogenesis. An important component of this system are the tight junctions, whose malfunction has been linked to the development of bowel cancer. Since some of the most important factors influencing the proper functioning of the intestinal barrier are diet and the absence of intestinal dysbiosis, correctly selected supplementation supported by appropriate dietary habits may favorably influence the development and course of cancer [44].

The data are mounting that the presence of the bacterium *A. muciniphila* in the human intestinal microbiome may significantly influence the effectiveness of cancer treatment, particularly for non-small cell lung and colon cancer [88,89,90]. Although not all of its mechanisms of action in the context of cancer have been understood, evidence suggests that this microorganism can exert a beneficial effect on immunity, with potentially important implications for the success of ongoing therapy [91]. Immunotherapy with anti-PD-1 and anti-PD-L1 antibodies is used in the treatment of some cancer types, such as non-small cell lung cancer, renal cell carcinoma, and melanoma. The efficacy of cancer immunotherapy depends on the ability of the host to produce tumor-antigen-specific Th1 cells that secrete IFNγ and cytotoxic effector T cells (Tc1) [92]. This raises the possibility for commensal gut bacteria having the ability to correct Th1/Tc1 immune responses, which can be defective. This offers considerable hope for the use of microbiota-focused interventions against both primary and secondary resistance to cancer immunotherapy [88,93].

After analyzing studies on the correlation of *A. muciniphila* and the body’s response to immunotherapy with an anti-PD-1 antibody, it was shown that the high presence of this bacterial species in the gut microbiota of lung or kidney cancer patients was associated with a positive body response to the administered therapy through increased mobilization of CCR9+, CXCR3+, and CD4+ T cells in tumor foci. It has been shown that commensal gut bacteria may have an effect on the regulation of endogenous immune stimulation and infiltration of T cells within the tumor environment. Interestingly, no such immune response was observed in patients whose gut microflora was deficient in *A. muciniphila*. In both cases, fecal microflora was collected from patients and transplanted into sterile mice. Here, too, the results clearly indicated increased T-lymphocyte activity within the tumor focus of mice with transplanted microbiota containing *A. muciniphila*. In contrast, mice that received transplants from patients who did not respond to treatment also showed no response to the administered therapy. An important fact is that after both transplantation of feces rich in *A. muciniphila* and after oral administration of the presented strain, a positive response of mouse organisms to anti-PD-1 antibodies was observed [91,94,95].

Another correlation of *A. muciniphila* with cancer treatment was noted for immune checkpoint targeting (ICI)—a therapy used in advanced stages of non-small cell lung cancer, melanoma, or renal cell carcinoma. The first key point was to prove that the anti-tumor effect of ICI immunotherapy depends on the presence of microorganisms in the gut. The researchers noted that patients who proceeded with ICI treatment responded significantly less well to the immunotherapy they received after the antibiotic treatment. Their life expectancy was shown to be reduced by 6.7 months (95% CI: 5.1–8.4). These observations were confirmed in prospective studies and large meta-analyses suggesting that the gut microbiota may play a key role in the immunostimulatory mechanism of action of ICI [88,96].

Prostate cancer (PCa) is one of the most common malignancies affecting men. Despite the successes achieved by ongoing immunotherapy in other types of cancer, there is still a need to develop a more effective therapeutic strategy for the treatment of PCa. To this end, a study was conducted to test the effect of the extracellular vesicles secreted by *A. muciniphila* (Akk-EVs) on developing PCa. During the study, isolated Akk-EVs were injected into immunocompetent mice every two days at a dose of 40 μg per mouse with implanted PCa. Mice were then monitored for immunophenotypic changes in cells, including CD8+ T-cell activation. Wound healing rates were also assessed to see how macrophages affect Akk-Ev-induced PCa cell proliferation and invasion. The body weight of the mice and their food and water intake were recorded throughout the study. After 13 treatments, it was shown that tumor proliferation was significantly slowed in the Akk-EV-treated mice compared to the control group. In addition, major organs such as the liver and kidneys were harvested for histopathology to assess potential systemic toxicity. The researchers’ conclusions were very promising; immunocompetent mice after Akk-EV administration showed a reduced prostate tumor burden with no observed toxicity to healthy tissues. The treatment resulted in increased granzyme B-positive (GZMB) and interferon γ-positive (IFN-γ) activity in CD8+ T-cells. An increased proportion of CD8+ T lymphocytes and the accumulation of more macrophages was found, with an increase in the number of M1 macrophages, which have tumor-fighting capacity, and a decrease in the number of M2 macrophages, which exhibit immunosuppressive effects. The macrophage growth environment conditioned by Akk-EV inhibited the proliferation and invasion of prostate cancer cells. Importantly, healthy cells tolerated the Akk-EV administered to the mice well. This study indicates a promising effect of *A. muciniphila* in immune therapy of prostate cancer [97].

At the turn of the year, it was noted that the gut microbiota may play a role in both the initiation and inhibition of colorectal cancer. A group of bacteria has been identified whose biofilm and potential to disrupt gut vascular barrier increases the risk of colon cancer [98] and augment liver metastases [99]. Such a relationship is shown, for example, by *Bacteroides fragilis* or *Fusobacterium nucleatum*. *F. nucleatum* also mediates resistance to chemotherapy and increases the bluntness of metastasis through activation of the autophagy pathway increasing chemoresistance of the tumor [100]. With this knowledge, the researchers decided to see if they could simultaneously isolate bacteria carrying a beneficial effect in the prevention and treatment of colorectal cancer. A study focusing on *A. muciniphila* was promising, as this bacterium showed a mitigating effect in mild and acute dextran sulphate sodium (DSS)-induced colitis by reducing levels of pro-inflammatory cytokines and improving host intestinal barrier function [101]. The first results on the efficacy and type of mechanisms of action of *A. muciniphila* in colorectal cancer showed that patients with colonic adenocarcinoma or colorectal cancer were characterized by a reduced abundance of this bacterium compared to healthy subjects. Interestingly, it was also observed that a significantly lower amount of *A. muciniphila* was found within tumor cells relative to adjacent normal cells [102].

Mice with induced spontaneous intestinal adenoma were divided into three different groups and given *A. muciniphila* (1 × 10^9^ CFU/d), *E. coli* (1 × 10^9^ CFU/d), or phosphate-buffered saline (PBS) for 14 days. They had previously received one week of antibiotic therapy. At week 12 after implementation of the intervention, the group treated with *A. muciniphila* significantly inhibited tumor growth compared to the PBS control group as assessed by tumor count (5.333 ± 0.7638 vs. 9.714 ± 1.04, *p* < 0.01), tumor volume (10% vs. 20% for tumors larger than 3 mm), and tumor burden (9.056 ± 1.621 vs. 15.11 ± 1.654, *p* < 0.05). In addition, tumors in the *A. muciniphila* had lower levels of the PNCA marker responsible for cell proliferation and an increased number of macrophages present in the tumor growth medium (TAM) type M1 compared to the PBS group (28.1% vs. 11.9%, *p* < 0.05). This suggests that *A. muciniphila* can stimulate M1-type TAMs to mount an immune response, which have beneficial therapeutic effects within colorectal cancer [102].

One study on the effect of *A. muciniphila* in the treatment of colorectal cancer indicates that its presence may be beneficial in first-line therapy with FOLFOX (oxaliplatin, fluorouracil, and calcium folinate), which in an experiment showed higher efficacy than the administration of oxaliplatin alone. It demonstrated that the abundance of *A. muciniphila* in patients receiving FOLFOX increased significantly. Most significantly, however, the increase in bacterial abundance in the intestine of patients was associated with increased antitumor efficacy of the administered preparation. A study was conducted in mice that had a significantly weakened and homogeneous intestinal microflora through broad-spectrum antibiotic therapy. They were then administered *A. muciniphila* (1 × 10^8^ CFU) every two days via gastric tube until the end of the study. The mice were divided into the following groups: control group, oxaliplatin model group, oxaliplatin-treated group, FOLFOX model group, and FOLFOX-treated group. Before administering the drug to the mice, it was verified, by collecting and testing fecal samples, that the graft of *A. muciniphila* passed successfully. Oxaliplatin and FOLFOX showed different effects on the composition and metabolism of the intestinal microflora. Oxaliplatin increased carbohydrate and nucleotide metabolism within the intestinal microflora, whereas FOLFOX increased carbohydrate and lipid metabolism. After analyzing the differences between the model groups and the groups receiving oxaliplatin and FOLFOX, it was shown that the most increased abundance of *A. muciniphila* was between the model group for FOLFOX and the FOLFOX-treated group. It also increased the anti-cancer effect of this therapy from 36% to 48% and the degree of tumor inhibition from 48% to 76% (*p* < 0.05). The above results indicate the efficacy of *A. muciniphila* in this type of colorectal cancer therapy. The group taking oxaliplatin alone had significantly worse results. Its antitumor effect was weaker than FOLFOX, so further research was devoted to the leading therapy. Although the study sounds promising, it is worth bearing in mind that it was conducted on mice that had previously received intensive antibiotic therapy to reduce the diversity and abundance of bacteria residing in the gut of the rodents [89].

In 2020, Wang et al. analyzed the effect of *A. muciniphila* injected to mice with sodium dextrasulphate followed by azoxymethane (DSSAOM) administration. The latter ones induce colitis, consequently leading to colorectal cancer. Mice were orally administered with either pasteurized *A. muciniphila* (1.5 × 10^8^ CFU) or Amuc_1100 protein (3 µg) as early as two weeks before the induction of inflammation and continued for a further 23 weeks during which the mice and their feces were systematically examined. At the same time, fecal samples were collected from patients with colitis and colorectal cancer. After completion of the study and analysis of the fecal samples, it was shown that in both DSS/AOM-infected mice and diagnosed patients, the concentration of *Akermanisa* in the intestine was reduced. It was noted that mice that received supplementation with pasteurized *A. muciniphila* or Amuc_1100 protein showed significantly later tumor development (*p* < 0.005) and also reduced tumor abundance (*p* < 0.05 for pasteurized *A. muciniphila* and *p* < 0.001 for Amuc_1100 protein) and tumor size (*p* < 0.01) at week 12 of the study. A reduced expression of tumor-associated markers γH2AX and Ki67 in colonic epithelial cells was also observed in mice treated with pasteurized *A. muciniphila* or Amuc_1100 protein. The above observations may suggest that supplementation of pasteurized *A. muciniphila* may inhibit excessive cell proliferation. The results are promising but should also be carried out fully on the human population [52].

**Figure 2:**
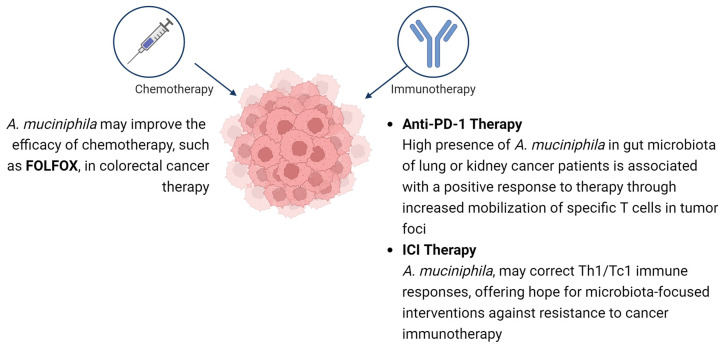
The potential benefits of *A. muciniphila* in cancer therapy. Created with BioRender.

In conclusion, *A. muciniphila* has a positive effect and contributes to the body’s response to certain agents administered in both immunotherapy and chemotherapy. Although the full mechanism of action has not yet been fully studied, we know that it plays a significant role in stimulating M1-type macrophages and increasing the number of CD8+ T lymphocytes. However, most of the studies have been carried out on animals, so further exploration and testing is needed. The summary of potential benefits of. *A. muciniphila* in cancer therapy is shown in Figure 2.

## 6. Neurodegenerative Diseases

Since the discovery of *A. muciniphila* in 2004 by Derrien et al. [19], evidence has emerged for the great potential of *A. muciniphila* and its outer membrane proteins in supporting the treatment of neuropsychological diseases. This evidence is supported by studies in animals and clinical trials [103]. The potential of *A. muciniphila* as a therapeutic support for neurological disorders such as Alzheimer’s disease (AD), multiple sclerosis (MS), amyotrophic lateral sclerosis (ALS), Parkinson’s disease (PD), or stroke has already been recognized. This suggests a viable role for *A. muciniphila* in normal brain function [36]. The interest of researchers from various backgrounds in conducting functional studies of the intestinal barrier under various conditions has led to the linking of these diseases to abnormal intestinal permeability, which is a sign of impaired intestinal barrier function. In PD and AD, a biomarker for assessing intestinal permeability is elevated levels of zonulin in the blood. It is therefore necessary to initiate measures leading to the restoration of a normal intestinal barrier and thus proper absorption of nutrients and fluids, preventing toxins and harmful bacteria from entering the body through the intestinal epithelium [104].

In mouse models of AD, a large decrease in *A. muciniphila* is observed [105], and this is associated with an impaired intestinal barrier [106]. These findings indicate the potential of *A. muciniphila* treatment in improving mucosal barrier dysfunction, metabolic dysfunction, and memory [107,108]. Mice on a high-fat diet showed a decline in cognitive and mental function, and this was associated with *A. muciniphila* depletion [109]. It is possible to make up for the deficit of this bacterium by, for example, a 16 week ketogenic diet, thus reducing the risk of neurodegeneration by improving the metabolic profile in young, healthy mice [110]. Beneficial effects of *A. muciniphila* supplementation whether by oral administration or dietary interference are observed in AD mouse models, compared to AD mouse models that do not receive this bacterium, and its fecal abundance decreases with age [103]. A mouse model of AD APP/PS1 fed a normal diet or a high-fat diet and treated with *A. muciniphila* (5 × 10^9^ CFU in 200 µL sterile PBS) by gavage daily for 6 months showed improvement in cognitive deficits and a reduction in amyloid-β (Aβ) protein levels [111]. Human studies have shown that patients with very mild to moderate AD have more *A. muciniphila* than the control group [112], while its levels are similar in people with mild cognitive impairment (MCI) and control subjects [113]. Studies on the beneficial effects of *A. muciniphila* on AD have been conducted relatively recently. However, direct evidence is still needed for validation, especially from human studies [36].

Postmortem findings in PD patients suggest that α-synuclein inclusions (a pathological feature of PD) may be transported from the gut to the brain via the vagus nerve [114], which raises the hypothesis that PD may begin in the gut. A growing number of recent studies have demonstrated alterations in the diversity of the gut microbiome in PD patients, which may cause inappropriate α-synuclein folding or disrupt enteric nervous system function [103]. Increased abundance of *A. muciniphila* was only found in mice with tetrahydropyridine (MPTP)-induced PD [115] and after treatment with rotenone [116]. However, *Akkermansia muciniphila* abundance did not increase in most mouse models of PD in other studies [117,118,119]. In contrast, studies of PD patients in clinical settings in different countries have shown increased abundance of *A. muciniphila* [120,121,122]. In a study that used three machine learning algorithms to analyze metagenomic results from 472 PD patients and 374 healthy controls, 22 bacterial families were identified that helped distinguish between controls and potential PD patients. Among the bacteria in these 22 families, *Akkermansia* showed high efficiency in distinguishing PD patients from controls [123]. These studies and findings present an important role for *Akkermansia* in the pathogenesis of PD. The increased abundance of *A. muciniphila* makes it a potential early biomarker of PD [124].

A study was conducted on the difference in microbial abundance between MS patients and controls, investigating how specific bacteria associated with MS modulate T-lymphocyte responses using in vitro and in vivo model systems. The results indicate that significantly increased abundance of *A. muciniphila* in MS patients is associated with a shift towards a pro-inflammatory T-cell profile that exacerbates or perpetuates the immune response. In an in vitro model, *A. muciniphila* extracts significantly increased the differentiation of peripheral blood mononuclear cells (PBMCs) into Th1 lymphocytes [125]. There are many studies confirming an increase in *A. muciniphila* abundance with the activation of the pro-inflammatory response in MS patients compared to controls [125,126,127]. In contrast, a reduction in *A. muciniphila* abundance in MS patients induced an anti-inflammatory immune response in the peripheral immune system [128]. Increased levels of *A. muciniphila* were also reported in a study in a mouse model of experimental autoimmune encephalomyelitis (EAE) compared with control mice [127]. Additionally, transplantation of the fecal microbiome from MS patients into mice induced a pro-inflammatory environment, which worsened the severity of EAE [125,129]. On the other hand, Cox et al. investigated a negative association between *A. muciniphila* abundance and disability and a positive correlation between *A. muciniphila* and brain volume, showing that the bacterium also plays a beneficial role. They sequenced the microflora in healthy controls, patients with relapsing–remitting multiple sclerosis (RRMS), and patients with progressive multiple sclerosis and correlated bacterial levels with clinical features of the disease, quality of life, and MRI brain atrophy. They colonized mice with *Akkermansia* from multiple sclerosis and induced experimental autoimmune encephalomyelitis. By testing RRMS patients with high levels of *Akkermansia*, they distinguished between three subtypes based on the 16S rRNA V4 sequence. They then isolated the bacteria on culture medium and sequencing identified the strains most similar to *A. muciniphila.* To illustrate the role of MS-derived *Akkermansia*, they colonized C57/BL6 mice with three strains and found that they all alleviated autoimmune encephalomyelitis (EAE). In addition, they measured immune responses and found that the *Akkermansia* sp. BWH-H3 strain reduced the number of RORγT-positive γδ T cells and IL-17-producing γδ T cells [130]. These findings support that increased levels of this battery in MS may be a beneficial compensatory response in the MS microbiome [130,131].

The studies in the case of MS and PD seem at first glance to be worrying and at odds with the previously described positive association between *A. muciniphila* abundance and health. Nevertheless, the following facts should be taken into account when analyzing the work described in this paragraph:

1. The studies described in MS and PD prove correlation, not causation.
2. In the course of neurodegenerative disorders, there is a significant increase in intestinal transit time [132] that leads to a proliferation of the microbiota [133].
3. The drugs used in the above-mentioned diseases can significantly affect the bacterial content, including *A. muciniphila* [134,135].
4. Bacterial contents in tests are given in relative abundances, which in practice means that their number (absolute value) is not necessarily higher [136].
5. Pasteurized *A. muciniphila* does not colonize the gut so does not induce changes in the composition of the microbiota [37].
6. No authority, including the EFSA, has denied the product’s placing on the market on the basis of these correlative reports alone [22].

*A. muciniphila* is negatively associated with amyotrophic lateral sclerosis (ALS), as evidenced by the progressively decreasing abundance of *A. muciniphila* in ALS mice [137]. Supplementation of pasteurized *A. muciniphila* in Sod1 transgenic mice (Sod1-Tg) with ALS improved motor function and brain atrophy through accumulation of nicotinamide in the central nervous system. Nicotinamide is associated with *A. muciniphila,* and its functions are to support dopamine production in neurons, neurotransmission, and normal neuronal cellular metabolism [108].

**Figure 3:**
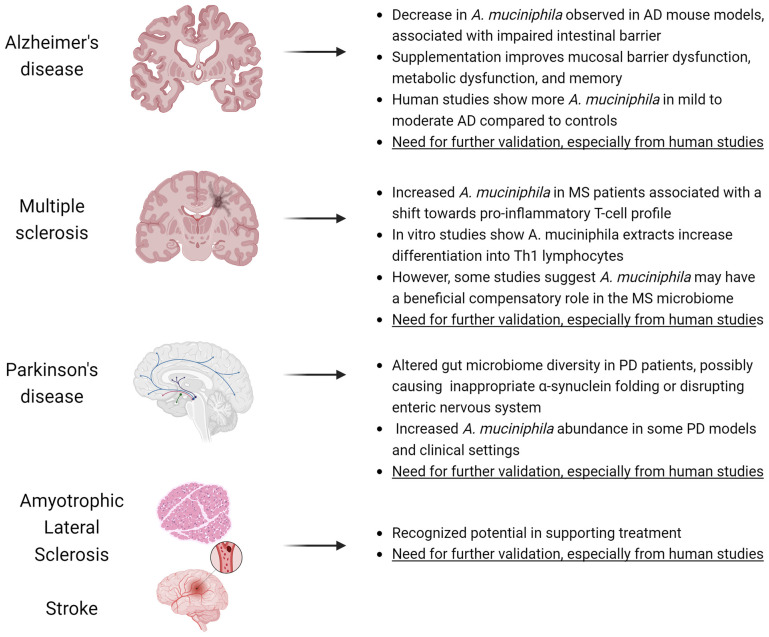
The role of *A. muciniphila* in neurodegenerative diseases. Created with BioRender.

Studies show that bacterial pneumonia is the leading cause of death after stroke. The selective movement of bacteria from the host’s gut microbiome to the lungs is an essential factor in causing this pneumonia. This leads to the conclusion that the intestinal mucosa and gut microbiota plays an important role in post-stroke mortality [138]. Studies have shown that stroke can be triggered by disturbances in the gut microbiota, which in turn can affect neuroinflammatory and functional indicators after brain injury. The efficacy of post-stroke treatment may be improved by fecal microbiota transplantation (FMT) [139]. An increase in fecal *A. muciniphila* levels has been noted in patients following ischemic stroke in clinical trials [140], leading to proposals to treat it as a microbiological marker [141]. There are other studies confirming that after stroke there is an increase in abundance in mouse models [138,142]. One of these studies analyzed the communities of different microorganisms in five gastrointestinal tract docs in mice after stroke: duodenum, jejunum, ileum, cecum, and colon. The finding of this study was that *A. muciniphila* changes its interaction profile after impaction towards activating beneficial bacteria, e.g., *Ruminococcus* spp., and inhibiting pathogenic bacteria, such as *Streptococcus* spp. and *Staphylococcus* spp. [138]. Thus, *A. muciniphila* contributes to reducing the migration of pathogens into epithelial cells and, consequently, into the lungs of stroke patients [138,143]. Different results were obtained in a fecal study of eight post-stroke humans and a 10 person control group, where the abundance of bacterial groups was compared. The abundance of *A. muciniphila* was reduced compared with the control group [144,145]. Still, the role of *A. muciniphila* in post-stroke patients remains unclear, so further studies, especially on larger study groups, are needed to determine this significance. Summary of the potential role of *A. muciniphila* in neurodegenerative diseases is presented in Figure 3.

## 7. Mental Illnesses

Research has shown that *A. muciniphila* may also play a role in neuropsychiatric disorders. Its direct influence is seen in the bacterium’s effect on the host microbiota–gut–brain axis. The bacterium itself and its metabolites play a role in alleviating neuropsychiatric symptoms by reducing intestinal imbalances, thereby contributing to improving the integrity and structure of the intestinal barrier, which translates into reduced inflammation [107]. Additionally, there is evidence in research that links stress response and psychiatric phenotypes to TLR signaling [146,147]. Research is scarce, mainly in animal models. One of the first studies was conducted in male C57BL/6N mice subjected to chronic stress (CRS), in which colitis was induced by administration of dextran sodium sulphate (DSS). The mice were then transplanted with *A. muciniphila* (1 × 10^9^ IU/mL) after reducing the diversity of the gut microbiota following the intervention. The study showed that CRS caused behavioral deficits but did not reduce the diversity of the gut microbiota. In addition, in CRS-treated mice, supplementation with *A. muciniphila* reduced the intensity of depressive disorders. Supplementation also improved histopathological indices—it improved mucosal barrier structure and function by increasing, among other things, the number of cup cells and also MUC-2 cells [148]. Another study also conducted in an animal model in male C57BL/6 mice aged 6–8 weeks sought to demonstrate the effect of CRS and *A. muciniphila* supplementation on depressive disorders. The control group received no supplementation, while two of the other groups were subjected to CRS, one of them with *A. muciniphila* supplementation for 3 weeks (5 × 10^8^ CFU/mL by oral gavage). The study showed that supplementation had a significant effect on the behavior of the mice, additionally reducing corticosterone levels and increasing dopamine and BDNF levels. However, no effect was observed on serotonin levels [149]. Another study sought to demonstrate how *A. muciniphila* supplementation would affect depressive and anxiety behaviors induced in animals by antibiotic use. Mice were supplemented with *A. muciniphila* for 3 weeks at 1.5 × 10^9^ CFU in one group, while the other group was given Amuc_1100 protein (100 μg/200 μL) daily. The study showed that supplementation reduced behavioral deficits induced by antibiotic therapy [150].

In a study by Mcgaughey et al. also conducted in mice subjected to social stress, there was a decrease in *A. muciniphila*, *Ruminococcus* spp., *Mollicutes* spp., *Paraprevotella* spp., and *Doreta* spp. However, an increase in the amount of *Oscillospira* spp., *Bacteroides* spp., and *Lachnospiraceae* spp. was observed. In addition, the amount of *A. muciniphila* was shown to correlate with behavior in mice [151]. Attempts were also made to demonstrate the effect of melatonin on modulating the composition of the gut microbiota in response to water stress and sleep deprivation. It was observed that in rodents, exposure to stress leads to a decrease in *A. muciniphila* and *Lactobacillus murinus*, but also to an increase in *Bacteroides masiliensis*. In addition, a decrease in melatonin concentration was observed, presumably as a mechanism of reduced bacterial abundance [152]. Sun et. al. report using broad-spectrum antibiotics in mice with induced depression. Pharmacotherapy resulted in increased anxiety and depressive behavior. In addition, reduced levels of 5-hydroxytryptamine (5-HT) were observed in both the mouse serum and hippocampus. Mice were supplemented with *A. muciniphila* (1.5 × 10^9^ CFU/200 µL) and Amuc_1100 (100 μg/200 µL) for 3 weeks. An alleviation of anxiety and depressive symptoms was observed. In addition, a normalization of BDNF expression in different brain areas and an increase in serum and hippocampal 5-HT was observed [150]. A study by Guo et al. sought to demonstrate a link between the occurrence of depression associated with alcohol abuse. After alcohol supply in rodents, there was damage to the intestinal barrier, as well as lipopolysaccharide (LPS) translocation, increased inflammatory response (increase in TNF-a and IL-1B), and decreased 5-HT levels. A 2 week supplementation with *A. muciniphila* (2.5 × 10^9^ CFU/200 μL) via oral gavage for 5 weeks resulted in an alleviation of depression-like behavior. Occludin, BDNF, and 5-HT increased, and LPS, TNF-a, IL-1b, and IL-6 decreased [153].

**Figure 4:**
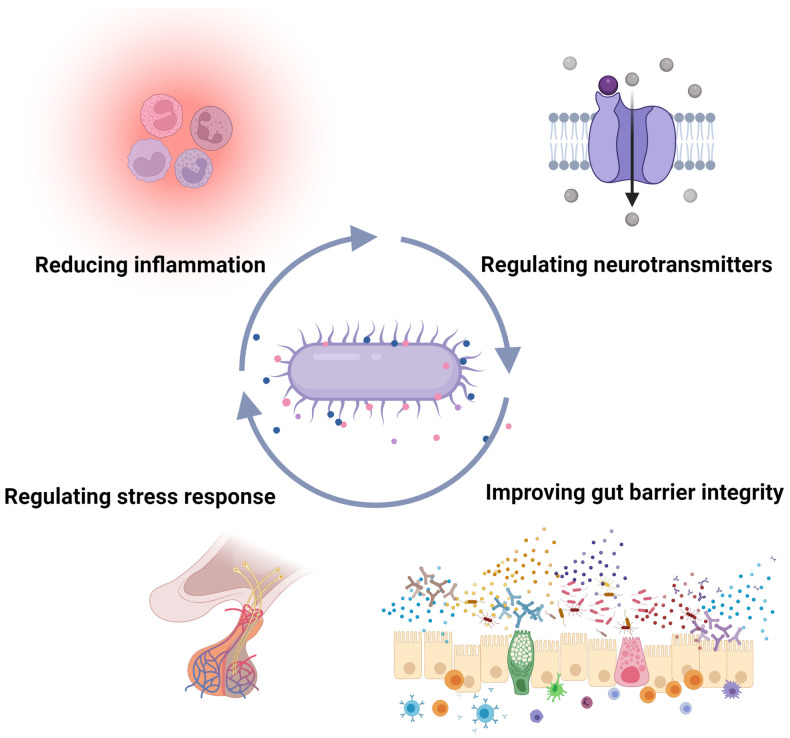
Modes of action of *A. muciniphila* in modulating mental health. Created with BioRender.

In autism spectrum disorders (ASD), the composition and function of the intestinal microbiota is disrupted. In a study by Zou et al., an increase in Bacteroidetes spp. and Firmicutes spp. was observed in patients with ASD. These patients also showed a reduction in *A. muciniphila*, *E. coli*, *B. fragilis*, and *H. parainflienzae*, among others [154]. In autism spectrum disorders, abnormalities in the composition and function of the gut microbiota are repeatedly highlighted, which translates into gastrointestinal dysfunction. In a mouse model of ASD, BTBRT + tf./j wished to test whether the use of a ketogenic diet would contribute to altering the composition and function of the gut microbiota. C57BL/6 and BTBR mice were fed standard food or a ketogenic diet for 10–14 days. After diet therapy, fecal samples and cecal sections were collected. The composition of the intestinal microbiota was altered in BTBR mice compared to control mice; in addition, the ketogenic diet reduced total fecal and colonic bacterial counts and increased *A. muciniophila* content [155]. A subsequent study conducted in a mouse model by Liu X. et al. sought to demonstrate the association of *A. muciniphila* supplementation and melatonin using a mouse model of valproic acid (VPA)-induced autism. It was shown that probiotic therapy (1 × 10^9^ cfu/mL; administrated by oral swabbing postnatal days 7–21) increased the activation of dopaminergic neurons during social interactions in the mouse model and also translated into metabolic changes [156]. The mechanism via which *A. muciniphila* confers benefits to mental health is depicted in Figure 4.

## 8. Modulating the Quantity of *A. muciniphila*

A number of studies have noted that dietary interventions significantly affect both the health of the subjects and the levels of *Akkermansia* spp. in the gut. Studies indicate that compounds such as polyphenols, resistant starch, FODMAP products (fermentable oligosaccharides, disaccharides and monosaccharides, and polyols) and individual products (e.g., red pitaya, whole grain barley, oat bran) can increase *A. muciniphila* abundance [82]. However, not all bran sizes work. An elegant study by Suriano et al. [157] aimed to assess the impact of different wheat bran fractions on the gut microbiota and their fat-binding capacity to understand their varying effects on metabolic and inflammatory disorders induced by a Western diet (WD) in mice. Over 8 weeks, mice on a WD were supplemented with arabinoxylan oligosaccharides (AXOS), crude wheat bran (WB), or reduced particle size wheat bran (WBs). AXOS altered gut microbiota composition, reduced *Clostridium* and *Turicibacter*, and increased *Bifidobacterium* and *Butyricicoccus*. Only WB increased *Akkermansia* levels by 11-fold. In a study by Walker et al., 500 mg resveratol was used for 5 weeks in patients with metabolic syndrome. The study did not show significant differences. However, considering the administration of resveratrol to Caucasians resulted in an increase in the number of *A. muciniphila* [158]. A study in the United States sought to demonstrate the effect of grape juice supplementation on metabolic parameters and the gut microbiota. In the study, C57BL/6J mice were fed a high-fat diet (HFD) supplemented with 1% Concord grape polyphenols. Compared to the control group, it was observed that the addition of polyphenols contributes to a reduction in the effects induced by the HFD diet (e.g., weight gain, inflammatory markers, glucose tolerance) and also improves intestinal barrier function by increasing the expression of genes including occludin. In addition, microbiome analyses showed a significant increase in *A. muciniphila* (predominantly in ileum and colon; in the jejunum to a smaller extent) and also a reduction in the ratio of *Firmicutes* spp. to *Bacteroidetes* spp. [82]. In a study conducted on 20 healthy volunteers, pomegranate extract (1000 mg) was used for 4 weeks. Data from the study were not presented for the whole population. Interestingly, the level of *A. muciniphila* depended on whether the subjects produced urolithin A. In this group, *A. muciniphila* levels were 47-fold higher after 44 weeks [159]. In a study conducted in Iran by Roshanravan, the subjects were divided into three groups. However, one of them received 600 mg/d of sodium butyrate, the second group received 10 g of inulin, while the third group received a combination of the two substances in the same amounts. The percentage increase in *A. muciniphila* abundance showed a significant increase in the group receiving sodium butyrate and inulin. A non-significant increase was obtained when both substances were co-administered [160].

Overall, plant-based diets, due to a large amount of fibers, predominantly were shown to increase the abundance of *A. muciniphila* [161,162].

## 9. Limitations

This narrative review has some limitations. Firstly, it is possible that our search strategy or systemic type may have missed some publications. Secondly, the included studies did not always contain explicit and detailed information on the form of *A. muciniphila* used. In addition, practically all studies are experimental in nature, and therefore it is difficult to draw conclusions about the practical use of *A. muciniphila* in medicine at this stage.

## 10. Conclusions

*A. muciniphila*, a prominent member of the gut microbiota, has demonstrated significant potential in influencing various aspects of host health, including metabolic, inflammatory, and neuropsychiatric conditions. This bacterium’s ability to improve intestinal barrier integrity, degrade mucin, and produce short-chain fatty acids positions it as a key player in gut health and beyond. Despite these promising findings, several challenges remain in translating these benefits into practical therapeutic applications.

Firstly, the stability and cultivation challenges of *A. muciniphila* have hindered its direct therapeutic use. However, recent advancements, such as the development of pasteurized *A. muciniphila*, have shown enhanced stability and potential health benefits, leading to its recognition as a novel food by the EFSA. This opens up new avenues for its application in managing metabolic diseases, including obesity and diabetes, as well as IBD. *A. muciniphila* has been linked to improved responses to immunotherapy and chemotherapy, particularly in non-small cell lung cancer and colorectal cancer. Its presence in the gut microbiota may enhance the efficacy of treatments like FOLFOX and immune checkpoint inhibitors, suggesting a synergistic role in cancer therapy.

Neurodegenerative and neuropsychiatric disorders also show a potential link with *A. muciniphila*. Studies in animal models indicate that *A. muciniphila* supplementation can improve cognitive function, reduce neuroinflammation, and alleviate depressive behaviors. However, the exact mechanisms and the extent of its impact on human neurodegenerative diseases require further investigation.

Despite the promising findings, it is important to note that most studies to date have been conducted in animal models. Human clinical trials are essential to validate these results and understand the practical applications of *A. muciniphila* in medicine. Additionally, dietary interventions and prebiotics that increase the abundance of *A. muciniphila* in the gut offer a practical approach to harnessing its health benefits.

In conclusion, *A. muciniphila* represents a promising target for therapeutic interventions across a range of diseases due to its multifaceted role in gut health and systemic disease modulation. Future research should focus on conducting extensive human trials and exploring dietary strategies to optimize its therapeutic potential.

## Figures and Tables

**Table 1:** The summary of advantages of using *A. muciniphila* as a therapeutic agent in gut diseases.

PROS	CONS
**Improves Gut Health**: Supplementation with *A. muciniphila* can enhance gut health by maintaining gut barrier integrity and reducing permeability.	**Reduced Levels in Disease**: In patients with IBD, *A. muciniphila* levels are significantly reduced, indicating a potential challenge in naturally maintaining its beneficial effects.
**Reduces Inflammation**: Oral supplementation of *A. muciniphila* or its protein Amuc_1100 reduces macrophage and cytotoxic T lymphocyte infiltration, thereby decreasing inflammation in colitis models.	**Research is Limited**: More research is needed to fully understand the efficacy and mechanisms of *A. muciniphila* in treating IBD and IBS.
**Protects Intestinal Barrier**: Increased colonization of *A. muciniphila* in the intestine elevates CREBH expression, which alleviates ER stress and reduces gut barrier permeability and blood endotoxemia.	**Variability in Abundance**: *A. muciniphila* abundance varies significantly among individuals and disease states, complicating its use as a reliable therapeutic agent.
**Potential in IBS Treatment**: Supplementation has shown potential in reducing visceral hypersensitivity, anxiety-like behavior, and pain sensations in IBS models.	**Effectiveness in Humans**: Although animal studies show promise, the effectiveness and safety of *A. muciniphila* supplementation in humans need further validation through clinical trials.
**Therapeutic Role in IBD**: Transplantation of intestinal microbiota increases *A. muciniphila* levels, correlating with improved clinical outcomes in IBD patients.	**Intestinal Mucosal Barrier Damage**: In cases of severe mucosal barrier damage due to pathogenic bacteria, the reduced levels of *A. muciniphila* pose a challenge in restoring its benefits.
**Inverse Relationship with Inflammation**: Studies confirm an inverse relationship between *A. muciniphila* abundance and inflammation, suggesting its protective role in gut diseases.	**Need for Targeted Therapies**: There is a need for targeted therapies to specifically enhance *A. muciniphila* colonization and activity in the gut for consistent therapeutic benefits.

**Table 2:** The impact of *A. muciniphila* intake on metabolic indices in animal models. EV—extracellular vesicles, LPS—lipopolysaccharide, GGT—gamma-glutamyl transpeptidase, AST—aspartate aminotransferase, LDH—lactate dehydrogenase, TG—triglycerides.

References	Model	Study Groups	Intervention	Survey Results
Wu et al. (2020) [83]	Animal	10-week-old male C57BL/6J mice orally fed	2 × 10^8^ per day; live or pasteurized *A. muciniphila*; 4 weeks	Induction of metabolism, reduction of body weight and improvement of body composition, reduction of insulin resistance, reduction of adipose tissue mass, induction of adipocytes, reduction of serum glucose after its oral administration, restoration of fat layer thickness after a high-fat diet.
Li et al. (2016) [79]	Animal	Apoe^−/−^ mice fed orally	5 × 10^9^ per day; live or pasteurized *A. muciniphila*; 9 weeks	Pasteurized *A. mucniphila.* Reduced fat gain, insulin resistance, and dyslipidaemia compared to mice supplemented with live A. muciniphila. Glucose tolerance comparable in both groups.
Zhao et al. (2017) [85]	Animal	Six-week-old pathogen-free mice on a low-carbohydrate diet	1 × 10^9^ for day, live *A. muciniphila*; 14 weeks;	Reduced weight gain and body fat, improved glucose tolerance and insulin sensitivity, reduced fatty-acid-related gene expression, reduced chronic low-grade inflammation, increased anti-inflammatory factors.
Chelakkot et al. (2018) [31]	Animal	6–8-week-old male mice C57BL/6	10 μg EV per day, 2 weeks	Reduced intestinal permeability, weight reduction; improved glucose tolerance.
Ashrafian et al. (2019) [32]	Animal	8-week-old male C57BL mice on a low-carbohydrate or high-fat diet	1 × 10^9^ live *A. muciniphila* per day; 10 mg protein/200 µL EV, 5 weeks	Significant weight loss in mice on a fat-rich diet, improved intestinal barrier integrity, improved glucose tolerance and lipid profile.
Everard et al. (2019) [78]	Animal	10-week-old male mice on a low-carbohydrate or high-fat diet	2 × 10^8^ per day; live *A. muciniphila*; 4 weeks	Reduction in diet-induced obesity; reduced appetite, body weight, and fat mass; reduced hyperglycemia and hyperinsulinemia.
Katiraei et al. (2019) [86]	Animal	9–13-week-old male E3L. CETP mice with an increased lipid profile	2 × 10^8^ per day; live *A. muciniphila*, 4 weeks	Reduction in body weight; reduction in plasma triglyceride and cholesterol levels.
Shin et al. (2019) [81]	Animal	8-week-old female C57BL/6 mice on a high-fat or low-carbohydrate diet treated with Akk growing on medium with (+) or without (−) mucilage addition	1 × 10^8^ live *A. muciniphila* per day, 4 weeks	*A. muciniphila* (−) attenuated the changes induced by the high-fat diet, reduced adipocyte hypertrophy and increased the proportion of small adipocytes, improved glucose levels and increased insulin tolerance, reduced LPS levels and inhibited diet-induced progressive intestinal inflammation.
Wu et al. (2020) [83]	Animal	8-week-old female C57BL/6 mice on a low-carbohydrate, high-fat diet	1 × 10^9^ live *A. muciniphila* per day, 10 months	Decreased weight gain, decreased appetite, increased expression of the anti-inflammatory factor IL-10.
Kim et al. (2020) [84]	Animal	5-week-old C57BL/6N mice on a high-fat, low-carbohydrate diet	1 × 10^8^–1 × 10^9^ live *A. muciniphila* per day, 10 weeks	No difference in weight gain, reduced TG and ALT levels in obese mice, reduced hepatic IL-6 expression in obese mice.
Lawenius et al. (2020) [87]	Animal	12-week-old female mice C57BL/6	Pasteurized *A. muciniphila* 2 × 10^8^ per day, 4 weeks	Reduced weight and fat gain, reduced incidence of T lymphocytes in the bone marrow.
Depommier et al. (2019) [37]	Human	32 people with overweight, insulin resistance, metabolic diseases	Pasteurized or live *A. muciniphila* 1 × 10^10^ per day, 3 months	Reduced plasma insulin levels and increased insulin sensitivity; reduced cholesterol, GGT, AST, LPS, LDH and serum creatine kinase; slight weight loss.

**Table 3:** Advantages of *Akkermansia muciniphila* supplementation in metabolic diseases.

Advantage	Proof
Enhancing gut barrier integrity	*A. muciniphila* improves gut barrier function, reducing permeability and endotoxemia.
Reducing inflammation	It decreases the levels of pro-inflammatory cytokines and markers, thereby alleviating systemic and gut inflammation.
Improving metabolic parameters	Supplementation has been shown to reduce insulin resistance, improve glucose tolerance, and lower plasma lipid levels.
Weight management	*A. muciniphila* has been linked to reduced body weight and fat mass, making it beneficial in obesity management.
Protecting against liver damage	It reduces liver enzymes (AST, ALT) and protects against liver fibrosis and NAFLD.
Supporting immune function	It enhances immune signaling, promoting a balanced immune response and reducing systemic inflammation.
Improving lipid profiles	Supplementation reduces cholesterol, LDL, and triglycerides levels, benefiting cardiovascular health.
Alleviating symptoms of metabolic syndrome	It has shown potential in reversing metabolic disorders like metabolic endotoxemia and associated conditions.
